# Clinical spectrum of osteomalacia: an atypical presentation

**DOI:** 10.11604/pamj.2025.52.142.49126

**Published:** 2025-12-04

**Authors:** Mahesh Sharma, Sourabh Deshmukh

**Affiliations:** 1 Department of Kayachikitsa, Mahatma Gandhi Ayurveda College, Hospital and Research Center, Salod (H), DMIHER (Datta Meghe Institute of Higher Education and Research, Wardha), Maharashtra, India

**Keywords:** Osteomalacia, osteopenia, tibia fracture

## Image in medicine

Osteomalacia is a metabolic bone disorder characterized by defective mineralization of the osteoid matrix, primarily due to vitamin D deficiency, calcium, or phosphate imbalance. This case highlights the classical clinical and radiological presentation of osteomalacia. A 45-year-old female presented with a history of persistent pain in the right leg for the past two years. She had a known history of hypertension and was on Telma 20 mg once daily. She also reported a previous fall from a bike, which resulted in a tibial fracture. She was admitted to the orthopedics department at that time and underwent surgery. Laboratory investigations revealed low vitamin D levels (8 ng/mL) and reduced calcium levels (1.80 mmol/L), and the patient was diagnosed with osteomalacia. She now presents for the management of persistent pain and generalized bone weakness. A radiographic evaluation (X-ray) demonstrated an implant in the right tibia, and the bones appeared markedly osteopenic and fragile. Laboratory investigations revealed vitamin D levels (25 ng/mL) and calcium levels (2.34 mmol/L). The patient was managed with local massage using Balashwagandha Taila, Tab. Amyron (1 tablet twice daily), Cap. Uprise D3 60,000 IU (once weekly), and Tab. Shallaki (1 tablet twice daily) for two months, which resulted in a significant reduction of pain. This case underscores the importance of correlating clinical findings with radiological features for the timely recognition and management of osteomalacia.

**Figure 1:**
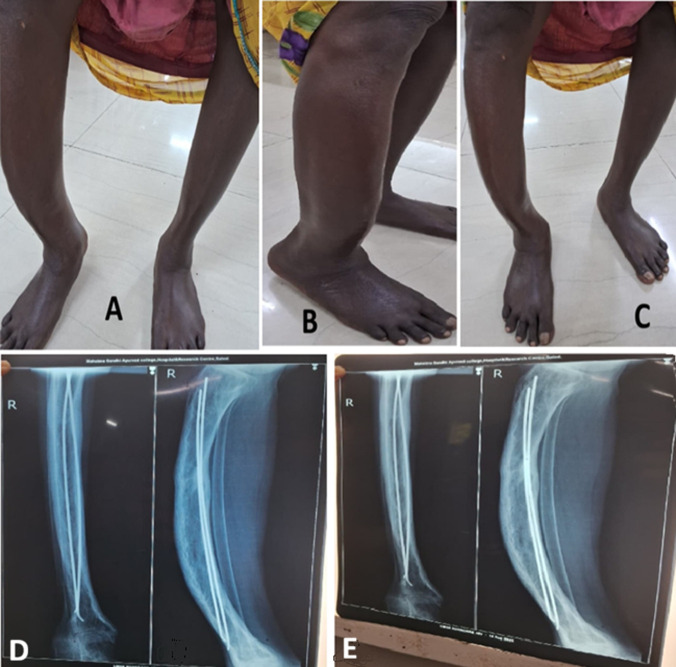
A) shape of the right leg from the medial aspect; B) shape of the right leg from the lateral aspect; C) shape of the right leg from the anterior aspect; D, E) radiograph X-ray of the right leg with an implant

## Available online at

https://www.panafrican-med-journal.com//content/article/52/142/full

